# Bridging the BCI illiteracy gap: a subject-to-subject semantic style transfer for EEG-based motor imagery classification

**DOI:** 10.3389/fnhum.2023.1194751

**Published:** 2023-05-15

**Authors:** Da-Hyun Kim, Dong-Hee Shin, Tae-Eui Kam

**Affiliations:** Department of Artificial Intelligence, Korea University, Seoul, Republic of Korea

**Keywords:** brain-computer interface, electroencephalogram, motor imagery, BCI illiteracy, style transfer, convolutional neural network

## Abstract

**Introduction:**

Brain-computer interfaces (BCIs) facilitate direct interaction between the human brain and computers, enabling individuals to control external devices through cognitive processes. Despite its potential, the problem of BCI illiteracy remains one of the major challenges due to inter-subject EEG variability, which hinders many users from effectively utilizing BCI systems. In this study, we propose a subject-to-subject semantic style transfer network (SSSTN) at the feature-level to address the BCI illiteracy problem in electroencephalogram (EEG)-based motor imagery (MI) classification tasks.

**Methods:**

Our approach uses the continuous wavelet transform method to convert high-dimensional EEG data into images as input data. The SSSTN 1) trains a classifier for each subject, 2) transfers the distribution of class discrimination styles from the source subject (the best-performing subject for the classifier, i.e., BCI expert) to each subject of the target domain (the remaining subjects except the source subject, specifically BCI illiterates) through the proposed style loss, and applies a modified content loss to preserve the class-relevant semantic information of the target domain, and 3) finally merges the classifier predictions of both source and target subject using an ensemble technique.

**Results and discussion:**

We evaluate the proposed method on the BCI Competition IV-2a and IV-2b datasets and demonstrate improved classification performance over existing methods, especially for BCI illiterate users. The ablation experiments and t-SNE visualizations further highlight the effectiveness of the proposed method in achieving meaningful feature-level semantic style transfer.

## 1. Introduction

Brain-computer interface (BCI) is a core technology that establishes a direct communication pathway between the human brain and an external device (Nicolas-Alonso and Gomez-Gil, 2012; Chaudhary et al., 2016; Jeong et al., 2022). Electroencephalography (EEG) has been actively used for current BCI systems in order to record the brain signals, due to its non-invasive, simple operation, relatively low-cost, and high temporal resolution (Lotte et al., 2007; Abiri et al., 2019). Over the last few decades, several neurophysiological studies (Decety, 1996; Munzert et al., 2009; Leeuwis et al., 2021) have shown that during motor imagery (MI), there are increased connections between sensorimotor areas in the human brain. In other words, MI can activate similar neural activities to those involved in actual motor movement (Case et al., 2015), and therefore it is feasible to identify the MI intention of the user by examining the EEG signals that exhibit unique patterns for each movement type.

In the MI-BCI paradigm, the user is instructed to imagine performing a specific body movement, such as the left hand or right hand, without actually executing the movement. During the MI period, the brain produces characteristic patterns of event-related desynchronization (ERD) and event-related synchronization (ERS), which can be observed in specific EEG frequency bands (Neuper et al., 2006; Pfurtscheller et al., 2006). Specifically, it is well-known that different ERD/ERS patterns are associated with different types of MI tasks, e.g., mu and beta frequency bands are reactive to imagined hand movements (Jeon et al., 2011). In this respect, EEG-based MI-BCI systems can be developed by using the ERD/ERS patterns to translate the imagined movement for recognizing the user's intention. As a result, MI-BCI has become one of the most promising sub-fields in BCI research and extensively studied over the past several years (Hamedi et al., 2016; Deng et al., 2021; Jeong et al., 2022).

Despite the great potential of MI-BCI, there is one major challenge to be addressed before using it in a real-world BCI application. In the MI-BCI research, it is widely recognized that there are substantial individual differences in the capability to perform a given MI task (Ren et al., 2020). Specifically, individual differences refer to the natural variation in personality traits, cognitive abilities, motivation, and other innate characteristics among individuals. Several studies (Allison et al., 2010; Volosyak et al., 2011; Wriessnegger et al., 2020) have found that these individual differences can influence MI-BCI performance and level of success in performing MI tasks. For instance, individuals who have low levels of motivation or who have difficulty maintaining attention may struggle with MI-BCI training and may not achieve the desired performance (Thompson, 2019). In the context of MI-BCI research, these aforementioned individuals are considered to have BCI illiteracy, which refers to the lack of knowledge and proficiency in using a BCI system within a standard training period (Lee et al., 2019; Volosyak et al., 2020). Based on previous studies (Becker et al., 2022; Tibrewal et al., 2022), around 15–30% of BCI users fail to produce the desired EEG patterns in order to control a BCI device accurately. Moreover, few studies (Zhang et al., 2020; Wang et al., 2021) have reported that BCI illiteracy subjects typically achieve classification accuracy below 70% and decrease the average performance of all subjects. This inability derived from BCI illiteracy can limit the applicability of the BCI system to be used by individuals who do not have specialized BCI knowledge or training. Thus, understanding and addressing BCI illiteracy is one of the major challenges for advancing the development of BCI technology (Ahn and Jun, 2015; Thompson, 2019).

In order to tackle the BCI illiteracy problem, various machine learning (ML)-based approaches (Vidaurre and Blankertz, 2010; Vidaurre et al., 2011; Tao et al., 2022) have been developed. One such approach is co-adaptive learning (Vidaurre and Blankertz, 2010), which uses the ML algorithm, i.e., linear discriminant analysis (LDA), to help users achieve closed-loop feedback. During the feedback process, both the user and the ML algorithm adapt to each other, thereby improving the overall performance of the BCI system. Another ML-based approach is based on multi-kernel learning (Tao et al., 2022) that aims to make the distribution of features closer to each other, while maximizing the divisibility of categories. Despite the reasonable performance achieved by ML-based approaches, they often rely on heuristic statistical reasoning and assumptions such as linear separability (Medin and Schwanenflugel, 1981) and same feature space assumption (Girolami, 2002). Moreover, these ML-based approaches may suffer from high computation costs when dealing with high dimensional data due to the curse of dimensionality (Bach, 2017). The EEG data are considered as inherently high-dimensional because they are typically collected using multiple electrodes with various spatial and temporal features. Thus, ML-based approaches are even more susceptible to these high-dimensionality issues.

Recently, a number of deep learning (DL)-based approaches (Tan et al., 2018; Gao et al., 2020; Zhao et al., 2020; Jeon et al., 2021; Sun et al., 2022) have been applied to the BCI illiteracy problem and achieved better performance compared to conventional ML-based approaches. In particular, deep transfer learning based on domain adaptation (Tan et al., 2018; Zhao et al., 2020; Jeon et al., 2021) has gained attention due to its capability to extract common feature representations. Specifically, Zhao et al. (2020) proposed a deep representation-based domain adaptation (DRDA) method that learns significant domain invariant features from multiple subjects (source domain) and uses that information to improve the performance on a single subject (target domain). Jeon et al. (2021) further developed it by utilizing mutual information to estimate the relevance of features and then extracting subject-invariant feature representations that are relevant to the classification task.

Even though these above-mentioned domain adaption methods have attained promising results, there are some disadvantages in their real-world BCI applications. First of all, these methods require a significant amount of labeled data in order to achieve good performance because they need to extract the common domain-invariant representations from multiple subjects (Sun et al., 2022). This can be problematic in BCI scenarios where large datasets do not exist or labeled data from multiple subjects are expensive to obtain in terms of time and cost. Second, they may suffer from negative transfer due to the large distributional discrepancy when extracting the common representation from multiple subjects (Jiménez-Guarneros and Gómez-Gil, 2021). Specifically, the negative transfer refers to a phenomenon that occurs when transferred knowledge or information from the source domain hinders the performance of the classifier on the target domain instead of improving it (Cao et al., 2018). Third, in real-world BCI applications, the EEG data from different domains vary substantially due to intra- and inter-subject variability (Saha and Baumert, 2020) that involves physiological noise level (Sanei and Chambers, 2013), signal quality (Ball et al., 2009), or emotions (Zhao et al., 2021). Therefore, these complex domain shifts derived from intra- and inter-subject variability can make it very difficult to find common domain-invariant feature representations across multiple subjects (Saha and Baumert, 2020). Hence, conventional domain adaption methods may fail to extract useful information from the source domain because of the large discrepancy within multiple sources. Besides, the EEG data of most BCI datasets are recorded over multiple sessions. So this session-to-session variability makes it even more difficult to construct a robust classifier across multiple subjects over multiple sessions.

Style transfer is also one approach for transferring between these domains. Traditional style transfer methods directly processed the image's content by applying various filters, machine learning, or probabilistic models (Ma et al., 2020). Gatys et al. (2015b, 2016a) proposed neural style transfer using convolutional neural networks, causing a paradigmatic revolution in the field, and this approach to neural style transfer became mainstream. Moreover, feedforward neural net-based approaches or iterative optimization methods (Isola et al., 2017; Yu et al., 2019) were proposed. These methods using style loss and content loss, effectively transfer the texture of a style image to a content image. However, they still have the limitation that they are mainly effective for the visual style and content. This is an important restriction because the nature of EEG data is such that differences are not visible.

To address these inter-subject variability issues, Sun et al. (2022) proposed another style transfer approach for the EEG classification tasks. Specifically, they introduced a subject transfer neural network (STNN) that directly transforms the data distribution from BCI-illiterate subject into BCI-expert, known as “golden subject”, by utilizing a subject-to-subject transfer approach. More precisely, the STNN model aims to learn a one-to-one style transfer between the golden subject (source domain) and the BCI-illiterate subject (target domain) without using any domain discriminator nor explicit regularizers. During training, the STNN uses only classification loss and perceptual loss, which compares the feature differences between source and translated target domains in order to facilitate the style transfer process. However, there are some limitations and drawbacks to the STNN. The main problem is that they only focus on transferring the style of the source domain and do not manage to preserve any content information from the target domain. Therefore, they may have difficulties in generating diverse and plausible data distributions containing desired content information from the target domain.

In this study, we first transform the high-dimensional EEG data into an image by using a continuous wavelet transform technique, and then we transfer the class-discriminative style of the source domain (BCI expert) to the target domain (BCI illiterates). In particular, we introduce a modified content loss to preserve the sementic content information of the target domain even after the style transfer process. Because the translated image of the target domain obtains class-discriminative characteristics from the source domain, we can improve the classification performance by leveraging the source classifier trained on the source domain data. Hence, our proposed method can alleviate the BCI illiteracy problem.

The main contributions of our proposed method are as follows:

1. To tackle the BCI illiteracy problem, we propose a subject-to-subject semantic style transfer network (SSSTN) that allows for effective and seamless interaction between users and the BCI system.
2. Unlike previous approaches that mainly focus on visible information, we introduce the semantic-aware style transfer loss function consisting of (i) a content loss to preserve the semantic identity information of the target domain, (ii) a style loss to transfer the semantic texture information of the source domain, and (iii) a semantic loss to further improve the classification performance.
3. By utilizing a subject-to-subject transfer strategy that performs a one-to-one mapping from the target domain to the source domain, our proposed method demonstrates high data efficiency, requiring only labeled data from a single subject in each domain dataset.
4. Our proposed method facilitates the construction of an ensemble classifier by integrating two subject-dependent classifiers—one from the target domain and the other from the source domain. This ensemble approach enables the fusion of diverse feature representations, resulting in a more robust classification model.

Our proposed method is evaluated on the BCI Competition IV-2a and IV-2b datasets, with experimental results showing that SSSTN outperforms other competing approaches in mean accuracy, particularly for BCI illiterates. Additionally, we performed an ablation study to assess the efficacy of each component within our proposed method. We also conducted ablation studies and visualization using t-SNE (Van der Maaten and Hinton, 2008) to evaluate the efficiency of each component within the proposed method. The experimental results demonstrated that the proposed method achieved meaningful feature-level semantic style transfer results.

## 2. Materials and methods

### 2.1. Dataset and preprocessing

#### 2.1.1. Dataset description

In this work, we used two publicly available benchmark datasets, namely BCI Competition IV-2a (Brunner et al., 2008) and 2b (Leeb et al., 2008), to evaluate our proposed method. The BCI Competition IV-2a dataset consists of EEG recordings from 9 healthy subjects, each performing four-class motor imagery tasks involving left hand, right hand, both feet, and tongue movements. For each subject, there were two separate EEG recording sessions that took place on different days. In each session, there were 72 EEG trials for each motor imagery task, resulting in a total of 288 EEG trials per subject. Each EEG trial lasted for 6 s, starting with a fixation cross, a cue followed by the motor imagery task. The EEG signals were recorded using 22 Ag/AgCl electrodes on the scalp in the 10-20 system (Homan et al., 1987), and the EEG data was sampled at 250 Hz. The BCI Competition IV-2b dataset comprises MI task experiments for two classes (right- and left-hand movements). The competition's objective was to classify MI tasks using EEG signals recorded from C3, CZ, and C4 channels. A total of 9 subjects participated in the experiment, with five sessions recorded for each subject. In each session, we used 60 trials for each motor imagery task, resulting in a total of 120 EEG trials per subject. Otherwise, the sampling rate and recording method are the same as for the BCI Competition IV-2a.

#### 2.1.2. Data preprocessing

For each trial, we obtained 4.5 s of EEG data by including 0.5 s prior to the start cue and 4.0 s after it (Schirrmeister et al., 2017). We then applied a bandpass filter ranging from 0.5 to 40 Hz and utilized exponential moving standardization to preprocess the raw EEG data. As a result, the preprocessed EEG signals consisted of a total of 1,125 time points (4.5 s × 250 Hz sampling rate) from 22 electrodes for the BCI Competition IV-2a dataset.

In this study, we decided to use a wavelet transform (Rioul and Duhamel, 1992) to transform the EEG data into images in order to use it as input data for our proposed SSSTN. Note that the use of wavelet transform is advantageous in our study for several reasons. Firstly, it allows for the effective representation of EEG signals in a multi-scale and multi-resolution manner, and this richer representation helps the algorithm to work with more informative data for the style transfer process. Secondly, transformation to images allows a convolutional neural network (CNN) to capture spatial-spectral-temporal features from multiple EEG representations from different electrodes, making it easier to perform semantic style transfer. Thus, after data preprocessing, we employed the continuous wavelet transform (CWT) technique (Rioul and Duhamel, 1992) to translate a one-dimensional signal from the temporal domain to a two-dimensional signal in the temporal-spectral domain. Specifically, the application of CWT to the EEG signal yielded a two-dimensional matrix, commonly referred to as a scalogram. Note that this scalogram matrix comprises the absolute values of the wavelet coefficients at different wavelet scales for the given EEG signals. Hence, this matrix can provide a detailed representation of the EEG signals in the temporal-spectral domain. By treating the scalogram matrix as an image, the 2D-CNN can be employed to extract spatial-spectral-temporal features and classify the MI tasks. Mathematically, the EEG signal *x*(*t*) can be transformed by CWT operation such as follow:


(1) $$\begin{array}{l} X_{w}\left(a,b\right)=\frac{1}{|a{|}^{1/2}}\overset{\infty }{\underset{-\infty }{\int }}x\left(t\right)\psi \left(\frac{t-b}{a}\right)dt, \end{array}$$

where *a*∈ℝ^+^ and *b*∈ℝ denote the scaling parameter for the spectral domain and the shifting parameter for the temporal domain, respectively (Rioul and Duhamel, 1992). Note that ψ(*t*) represents the *Morlet* wavelet (Grossmann and Morlet, 1984), which is one of the most widely used wavelet base functions because it provides good resolution in both temporal and spectral domains. To be more specific, the *Morlet* wavelet ψ(*t*) is expressed as follow:


(2) $$\begin{array}{l} \psi \left(t\right)=\text{exp}\left(-\frac{\beta^{2}t^{2}}{2}\right)\cos\left(\pi t\right), \end{array}$$

where β represents the admissibility condition that determines the balance between the spectral resolution and the temporal resolution of the wavelet *Morlet*. The images of each subject obtained through the CWT process are used as input data for the generator in the style transfer process.

### 2.2. Methods

**Figure 1:**
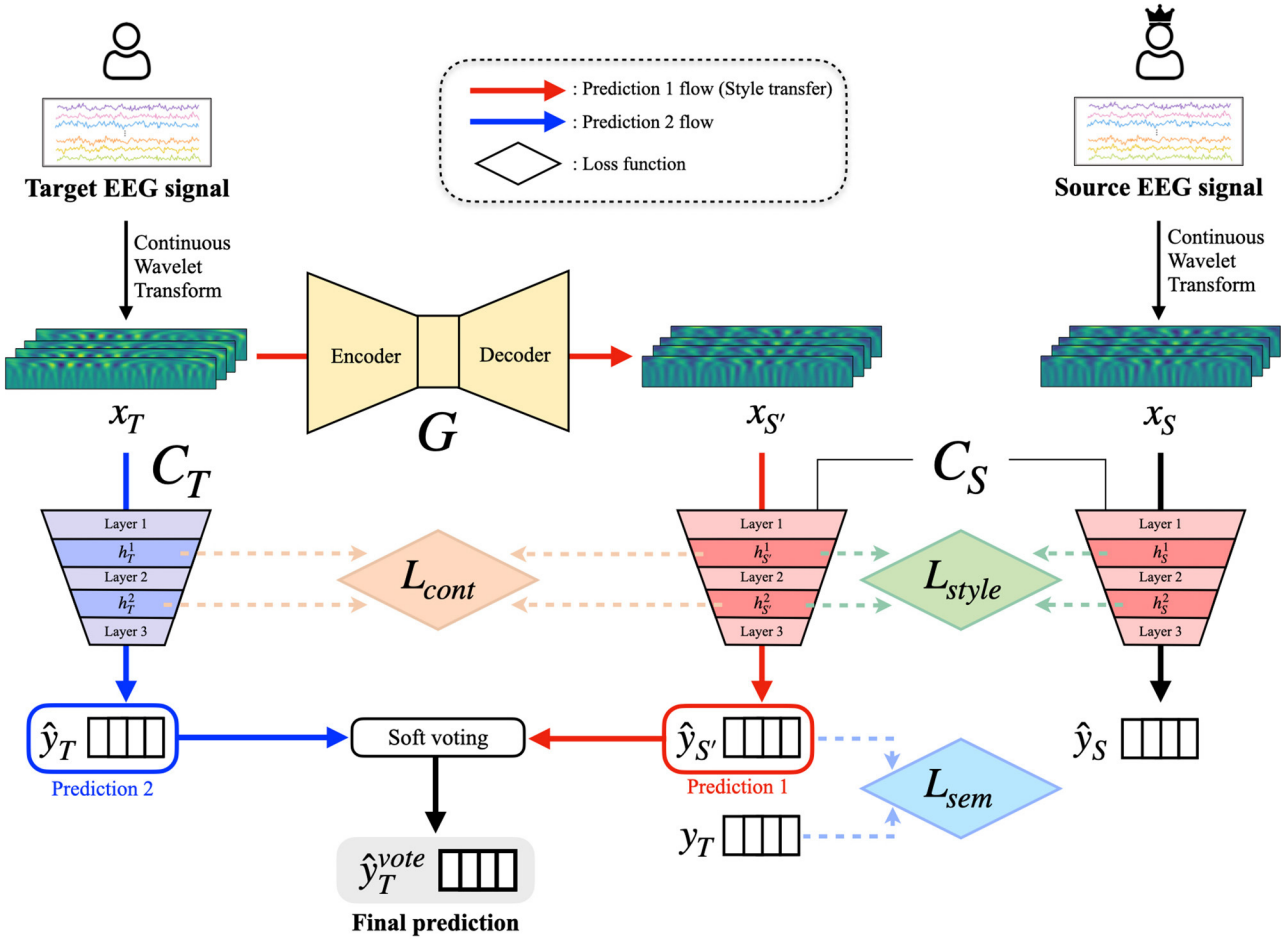
Overview of the proposed SSSTN framework. The SSSTN consists of three phases: (1) pretraining, (2) style transfer, (3) prediction and ensemble. The input variables, *x*_*T*_ and *x*_*S*_, represent the target and source subject data, respectively, while $x_{S^{\prime }}$ signifies the target data transformed by the generator *G*. During the pretraining phase, the classifiers *C*_*T*_ and *C*_*S*_ are pretrained using *x*_*T*_ and *x*_*S*_, respectively. In the style transfer phase, only *G* is trained. Content loss is computed using features $h_{T}^{l}$ and $h_{S^{\prime }}^{l}$ from the *l*-th convolutional layer after passing *x*_*T*_ and $x_{S^{\prime }}$ through *C*_*T*_ and *C*_*S*_, respectively. Style loss is calculated between $h_{S^{\prime }}^{l}$ and $h_{S}^{l}$ after passing $x_{S^{\prime }}$ and *x*_*S*_ through *C*_*S*_, respectively. Semantic loss is computed between the predicted label ${ŷ}_{S^{\prime }}$, which is obtained by passing $x_{S^{\prime }}$ through *C*_*S*_, and the ground-truth label *y*_*T*_. In the last phase, the final prediction is generated using a soft voting ensemble of ${ŷ}_{S^{\prime }}$ and ŷ_*T*_, where ŷ_*T*_ is the predicted label for *x*_*T*_ obtained from *C*_*T*_.

Here, we propose a Subject-to-subject Semantic Style Transfer Network (SSSTN) for tackling the BCI illiteracy problem in EEG-based MI classification tasks. Unlike conventional style transfer networks, our SSSTN not only performs style transfer but also transfers and generates class-discriminative data while taking into account the semantics of the input data. Since we transform an EEG signal into an image by using the CWT method, the stylistic differences between the source and target data may not be easily distinguishable as to be visible. Consequently, we think that existing style transfer approaches are not suitable for this task. To address this issue, we introduce a modified style loss and content loss that are tailored to this specific task of transferring semantic style and semantic content information in an effective manner. During the transformation of the target subject (especially, BCI illiterates) data into the source subject (BCI expert) data, our SSSTN effectively transfers and preserves the underlying semantic style and content information at feature-level in order to ensure that the source subject classifier is able to accurately classify the transformed data. The overall flow of our proposed method is depicted in Figure 1. The training process of SSSTN can be divided into three phases: (1) Pretraining, (2) Style Transfer, and (3) Prediction and Ensemble. Detailed explanations of each phase are presented below.

#### 2.2.1. Pretraining

**Figure 2:**
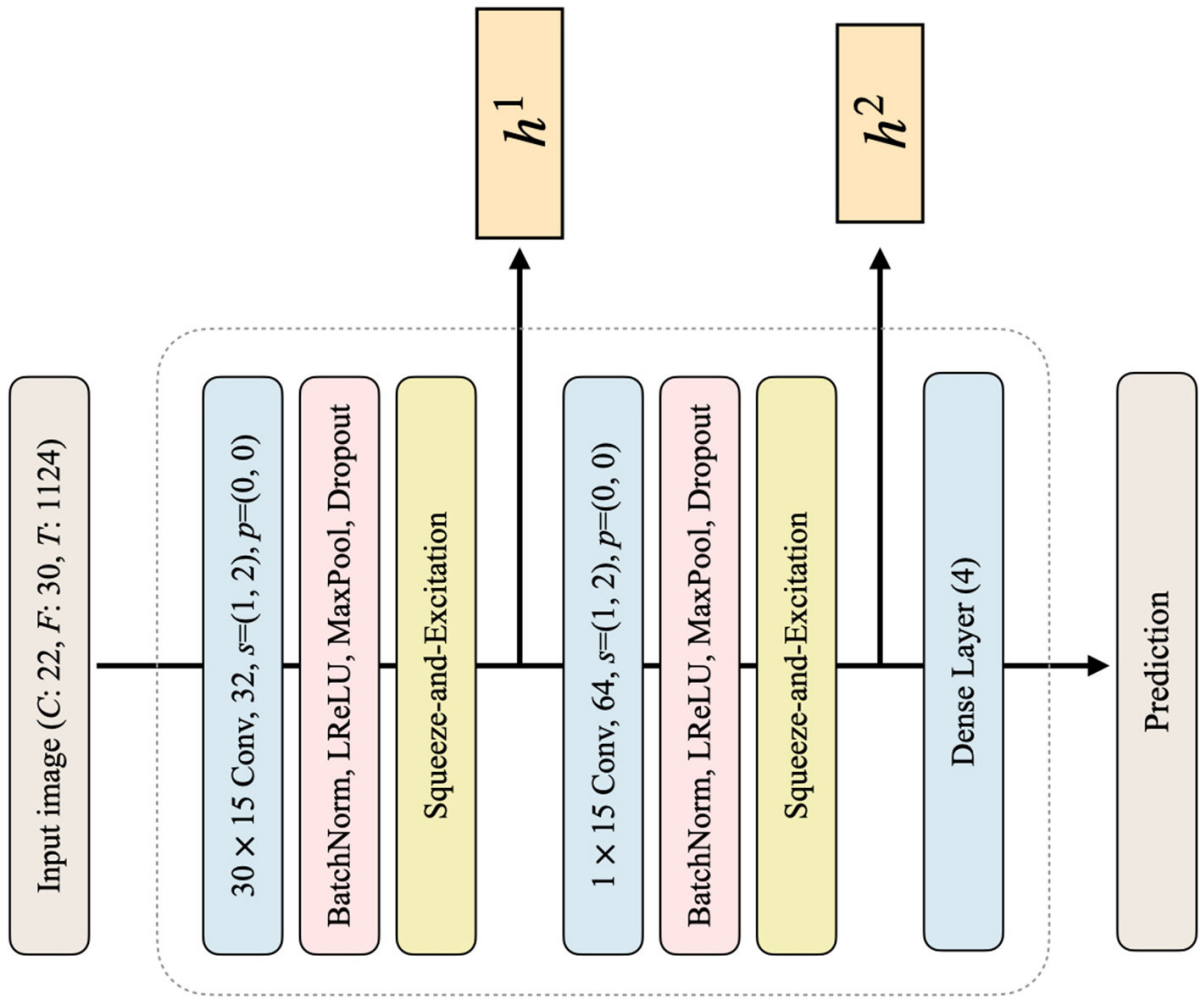
The architecture of the classifier *C* in the proposed framework. The dimensions of the input image are denoted by *C*, *F*, and *T*, corresponding to the number of channels, frequencies, and time points, respectively. The kernel size in each convolutional layer is followed by a number indicating the number of output channels. The variables *s* and *p* denote the stride and padding used in the convolutional layers, respectively. The activation function used in this architecture is LReLU, which stands for Leaky ReLU. The output features of the first and second convolutional blocks are represented as *h*^1^ and *h*^2^, respectively, and are utilized to calculate the loss. Finally, the number written in the dense layer indicates the dimension of the output feature, resulting in a prediction of size 4 at the end.

In the pretraining phase, we train a classifier for each subject using image data, i.e., obtained through the CWT method, and then perform a classification task under the subject-dependent scenario (subject-specific training). The primary objective of the pretraining process is to train the classifier, which can accurately classify input image data into one of the four distinct MI classes. The architecture of the classifiers utilized in this process is represented in Figure 2. The numerical values in the convolutional layer box represent the kernel size, number of output channels, stride, and padding, respectively. Note that the classifier architecture remains consistent across all subjects, comprising two convolutional layers and one dense layer. The convolutional layers are composed of batch normalization, LeakyReLU activation, max pooling operation, and dropout (Zhang et al., 2021). After each convolutional layer, a squeeze-and-excitation (SE) (Hu et al., 2018) module is employed. The SE module is responsible for capturing the important channel-wise relationships, which is crucial in EEG signals where interdependencies between channels are important, as each channel represents an electrode. After each SE operation, the feature *h*^*l*^ is used to calculate the loss in the subsequent step of style transfer, where *l*∈*L* = {1, …, *L*} denotes the *l*-th convolutional layer of the classifier *C*. Our proposed model utilizes a total of two convolutional layers, denoted as *L* = 2 in our case. After the two convolutional layers and the SE modules, the dense layer produces the prediction. The dense layer is a fully-connected layer that flattens the input features and then performs fully-connected operations, returning them as predictions for the four classes. Finally, classifiers are trained with the following classification loss:


(3) $$\begin{array}{l} L_{cls}=-\sum_{k=1}^{K}y^{\left(k\right)}\log{\hat{y}}^{\left(k\right)}, \end{array}$$

where *K* denotes the total number of classes. In our case, each classifier performs a 4-class classification task, which sets the value of *K* to 4. Additionally, during this stage, we identify the subject with the highest classification performance to serve as the source subject for style transfer, while designating all other subjects as target subjects.

#### 2.2.2. Style transfer

It is worth noting that the objective of the style transfer phase in our study differs from that of general style transfer approaches. In general style transfer, the style is typically represented by the texture or color of the image, while the content refers to the object depicted in the image. However, the semantic style and content in our study are not defined by visible textures, colors, objects, or general scenery. As a result, they may not be immediately apparent or easily recognizable in the image data. As previously mentioned, there exist substantial inter-individual differences in EEG characteristics within MI-BCI (Ren et al., 2020). Therefore, even if multiple subjects are trained with the same classifier structure, the features of each subject are represented in distinct feature spaces. Among these feature spaces, the feature space of the source subject with the highest classification performance, which we refer to as BCI experts, contains source features that are well-classified by the source classifier. It is reasonable to assume that this feature space is where the source classifier performs effectively. Thus, if the generator can accurately map the target features to the source feature space while retaining class-relevant feature representation, the resulting transformed features can be expected to be effectively classified by the source classifier. In this context, the class-relevant feature representation of the target feature is considered as the content that should be preserved during the transfer process, and the feature space where the source feature is located is treated as the style that should be applied to the target data.

**Figure 3:**
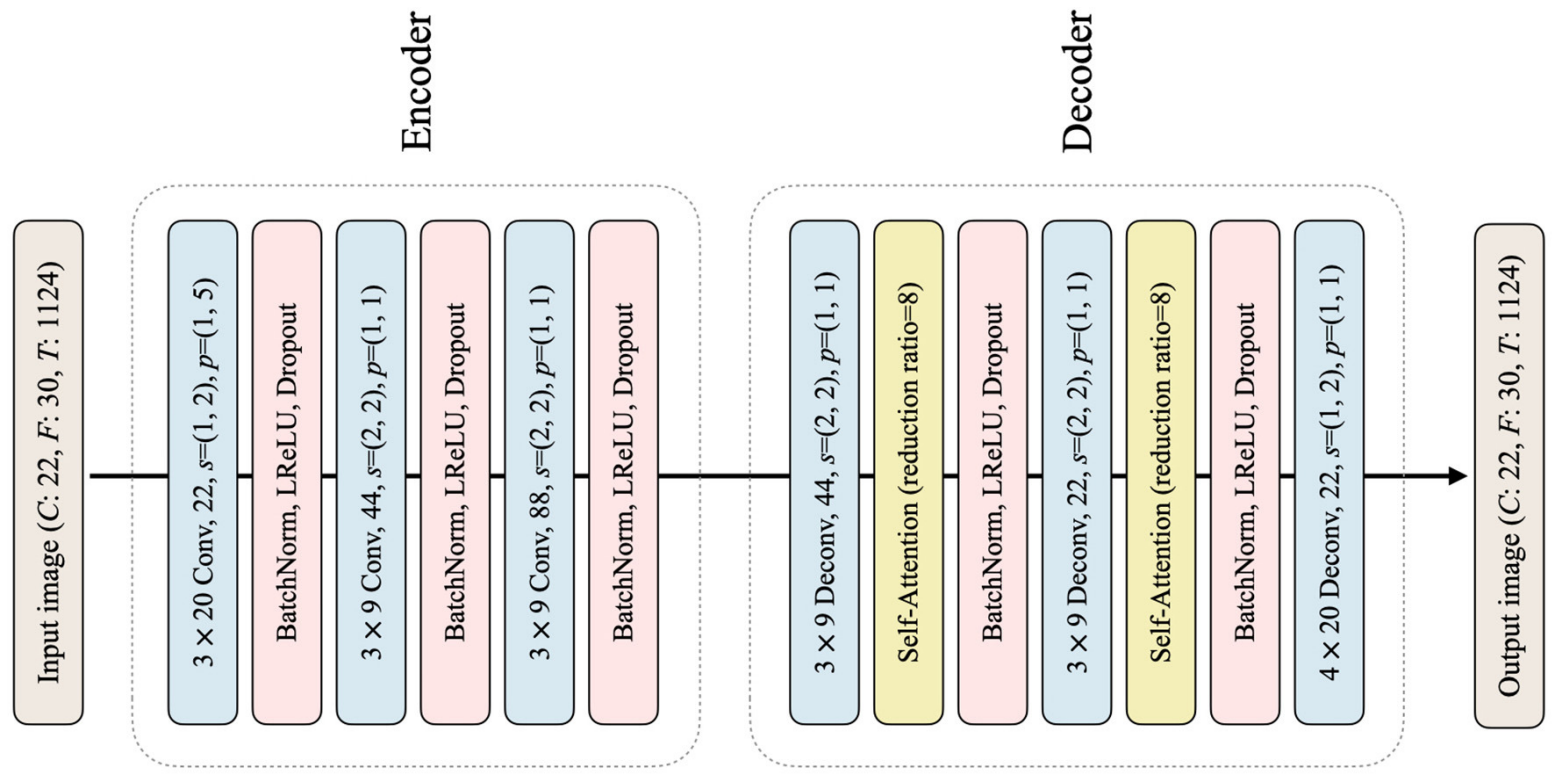
The architecture of the classifier *G* in the proposed framework is shown, with deconv representing the transposed convolutional layer. All other notations used are consistent with those shown in Figure 2.

In the style transfer phase, the generator *G* is responsible for subject-to-subject style transfer by transforming the target data from each target subject to align with the style of a single source data (in our specific case, subject 3 was selected as the source). The primary objective of *G* is to transform the input image in such a way that the resulting target data reflects the style of the source data at feature-level, while concurrently preserving the content of the target data. This is achieved by effectively mapping the target features to the source feature space, enabling the transformed features to be accurately classified by the source classifier. The architecture of *G* follows an encoder-decoder structure as illustrated in Figure 3. The encoder module consists of three convolutional layers, each with batch normalization, LeakyReLU activation, and dropout. The decoder module, on the other hand, consists of three transposed convolutional layers. Notably, the first and second transposed convolutional layers are followed by a self-attention layer (Vaswani et al., 2017; Zhang et al., 2019). The self-attention layer is incorporated into the generator to enhance its capability to selectively emphasize relevant features in the input data that are crucial for precise classification during the transformation of target data into source data (Sun et al., 2022). After each self-attention layer, batch normalization, LeakyReLU, and dropout are applied to the encoder. The output image generated by *G* is of the same size as the input image. It is noteworthy that during the entire style transfer process, the pretrained source classifier *C*_*S*_ and target classifier *C*_*T*_ remain fixed, while *G* is the only network being trained. The entire loss function of *G* consists of style loss, content loss, and semantic loss, which are combined to guide the network toward the desired outcome.

The features $h_{S^{\prime }}^{l}$ and $h_{S}^{l}$ are obtained by passing the transformed and source data, $x_{S^{\prime }}$ and *x*_*S*_, respectively, through the *l*-th convolutional layer of *C*_*S*_. The objective of the style loss is to align the feature distributions of the transformed target features $h_{S^{\prime }}^{l}$ and the style features $h_{S}^{l}$ in the feature space. This is achieved by measuring the discrepancy between the Gram matrices of $h_{S^{\prime }}^{l}$ and $h_{S}^{l}$ (Gatys et al., 2015b, 2016a). By minimizing the style loss, the distribution of $h_{S^{\prime }}^{l}$ becomes more similar to that of $h_{S}^{l}$. The style loss can be mathematically defined as follows:


(4) $$\begin{array}{l} L_{style}=\sum_{i=1}^{N}KL(\sigma \left(Gr\left(h_{S^{\prime }}^{l}\right)\right)\;\|\;\sigma \left(Gr\left(h_{S}^{l}\right)\right)),\\ \;Gr_{ij}^{l}=\sum_{m=1}^{M}h_{im}^{l}h_{jm}^{l}, \end{array}$$

where *KL* and σ represent the KL divergence and softmax activation function, respectively, while *Gr* stands for the Gram matrix and *N* represents the sample size. The ${Gr}_{ij}^{l}$ is the inner product between vectorized feature maps *i* and *j* in layer *l*, and the *M* indicates the total number of spatial locations in the feature map (Gatys et al., 2015a, 2016b). In order to achieve further semantic style consistency between target and source data, we employed the Gram matrix-based style loss (Gatys et al., 2015a) in conjunction with the Kullback-Leibler divergence (KL divergence) (Kullback and Leibler, 1951). The application of Gram matrices has become a widely adopted technique for matching second-order statistics between different feature activations in various studies (Gatys et al., 2015a, 2016b; Huang and Belongie, 2017). Building upon previous studies using Gram matrices, we utilize the KL divergence with a softmax function applied to the Gram matrix representations of $h_{S^{\prime }}^{l}$ and $h_{S}^{l}$ as the style loss. Of note, the KL divergence represents the distance between two distributions. By minimizing the style loss based on KL divergence between the Gram matrix representations of $h_{S^{\prime }}^{l}$ and $h_{S}^{l}$, the generator *G* can bring the distribution of $h_{S^{\prime }}^{l}$ closer to that of $h_{S}^{l}$, both of which are based on the feature space of *C*_*S*_. Consequently, our proposed approach results in $x_{S^{\prime }}$ being better classified by *C*_*S*_, thereby achieving improved style transfer performance.

Our proposed style loss serves as a regularization technique for the target data (*x*_*T*_) by guiding *G* to transform it in a direction that incorporates more of the semantic style of the source data (*x*_*S*_) (Huang et al., 2022). However, unilaterally transferring only style information can result in negative transfers. To address this issue, we introduce a content loss term that enforces the preservation of the semantic content in *x*_*T*_ before and after the transformation. The mathematical formulation of our content loss is provided below:


(5) $$L_{cont}=\frac{1}{N}\sum_{l=1}^{L}{\left(h_{T}^{l}-h_{S^{\prime }}^{l}\right)}^{2}\;,$$

where *N* represents the sample size again and the features $h_{T}^{l}$ are obtained from the *l*-th convolutional layer of the target classifier *C*_*T*_. The feature representation $h_{T}^{l}$ contains class-relevant information of the target image *x*_*T*_, and thus transferring it to $x_{S^{\prime }}$ ensures that *G* preserves the semantic content of *x*_*T*_ during the transformation.

The ultimate objective of SSSTN is to ensure that the generated image $x_{S^{\prime }}$ is correctly classified by *C*_*S*_. To achieve this, we use semantic loss, which is designed to minimize the classification error of $x_{S^{\prime }}$ in the MI classification task. Specifically, we compute the cross-entropy loss between the prediction ${ŷ}_{S^{\prime }}$ and true label *y*_*T*_ of *x*_*T*_. The resulting semantic loss is shown below:


(6) $$\begin{array}{l} L_{sem}=-\overset{K}{\underset{k=1}{\sum }}y_{T}^{\left(k\right)}\log{ŷ}_{S^{\prime }}^{\left(k\right)}, \end{array}$$

where *K* represents the total number of classes, which is 4 in our specific case. The total loss is defined as follows:


(7) $$\begin{array}{l} L_{total}=\alpha L_{style}+\beta L_{cont}+\gamma L_{sem}, \end{array}$$

where α, β, and γ are weighting factors for style, content, and semantic loss, respectively. Recall that our style loss and content loss do not directly enforce $x_{S^{\prime }}$ to match *x*_*S*_, but instead leverage *C*_*S*_ and *C*_*T*_ to measure the similarity at feature-level between the two. The goal of the style loss and content loss is to ensure that $h_{S^{\prime }}^{l}$ is mapped to a feature space that is similar to $h_{S}^{l}$, while preserving the class-relevant representation of $h_{T}^{i}$. Therefore, minimizing this total combined loss leads to $x_{S^{\prime }}$ having high classification performance on *C*_*S*_.

#### 2.2.3. Prediction and ensemble

After completing the style transfer phase, two predictions for *x*_*T*_ can be obtained. The first prediction, denoted as ${ŷ}_{S^{\prime }}$, is derived from *C*_*S*_ for $x_{S^{\prime }}$ that is generated by *G*. The second prediction, denoted as ŷ_*T*_, is derived from *C*_*T*_ for *x*_*T*_. Next, we employ a soft voting-based ensemble to obtain the final prediction by combining the two predictions obtained for *x*_*T*_. A soft voting-based ensemble is a well-established method of combining multiple classifiers by taking into account both individual decisions and probability values to assign data to a specific class (Sherazi et al., 2021). Soft voting has been demonstrated to yield better performance and results than hard voting since it utilizes an average of probabilities (Saqlain et al., 2019). As such, soft voting has been widely used in many BCI studies due to its potential to improve the classification performance (Mussabayeva et al., 2021; Tasnim et al., 2022; Mehtiyev et al., 2023). In addition, soft voting-based ensembles are effective in compensating for the weaknesses of individual classifiers and can achieve even better performance when combining classifiers trained on different features. The increased diversity in feature space is the key factor behind the performance improvement of ensemble classifiers, as it enhances their robustness to both inter- and intra-subject variability (Corsi et al., 2022). Due to the fact that *C*_*T*_ and *C*_*S*_ were trained on distinct datasets, namely *x*_*T*_ and *x*_*S*_, respectively, their feature spaces are different and complementary. Consequently, by leveraging both classifiers through an ensemble method, we can effectively utilize multiple feature spaces to enhance the classification performance. Mathematically, the final prediction ${ŷ}_{T}^{vote}$ is obtained by employing the soft voting-based ensemble method, as depicted below.


(8) $$\begin{array}{l} {ŷ}_{T}^{vote}={ŷ}_{S^{\prime }}+{ŷ}_{T}, \end{array}$$

where ${ŷ}_{S^{\prime }}$ and ŷ_*T*_ represent the predicted labels obtained from subject-specific classifiers *C*_*S*_ and *C*_*T*_, respectively. The final prediction is determined by selecting the class with the highest logit value in ${ŷ}_{T}^{vote}$. To sum up, our proposed style transfer approach leverages subject-specific classifiers to facilitate the use of the ensemble method, resulting in enhanced performance and classification accuracy.

## 3. Results

### 3.1. Competing methods

We evaluated the proposed SSSTN on accuracy by comparing it to the following competing methods on the BCI Competition IV-2a and the BCI Competition IV-2b datasets. For a more accurate and intuitive comparison, we categorized the competing methods into signal-based ([1]~[4]) and image-based ([5]~[8]), as shown in the Table 1.

[1] EEGNet (Lawhern et al., 2018) is tailored to exploit the unique spatial and temporal structure of EEG signals, combining temporal and spatial convolutions to capture relevant information. The model is lightweight and adaptive and has shown good performance for low computational cost in various BCI studies.
[2] DeepConvNet (Schirrmeister et al., 2017) focuses on exploiting the inherent spatial and temporal structure of the EEG data by employing specialized convolutional layers that capture the intricate patterns within the signals. Furthermore, the authors introduce a visualization technique that allows for the inspection and interpretation of the learned representations in the context of the underlying neural processes.
[3] DRDA (Zhao et al., 2020) proposes a deep representation-based domain adaptation approach to address non-stationary EEG classification challenges. The methodology focuses on learning transferable and discriminative representations by bridging the gap between source and target domains, while simultaneously preserving the class-discriminative information.
[4] MI (Jeon et al., 2021) presents a method that leverages mutual information for deep representation learning in brain-computer interface (BCI) applications. The proposed approach aims to achieve subject-invariant and class-relevant representations by optimizing mutual information between the learned features and class labels, while minimizing subject-related information. This method facilitates enhanced generalization across subjects and increased classification performance in BCI tasks.
[5] CWT-CNN (Mahamune and Laskar, 2021) employs two-dimensional images, generated through continuous wavelet transform (CWT) filter bank decomposition of pre-processed EEG data using the multi-class common spatial pattern (CSP) technique. These 2D images serve as the basis for training a convolutional neural network (CNN), enhancing classification accuracy.
[6] SE-CNN inspired by Zhang et al. (2021) is designed as the backbone classifier for our SSSTN in this study. It consists of two convolutional layers and SE modules, as described above. Therefore, the performance of SE-CNN corresponds to the version of the pretraining phase of SSSTN.
[7] STNN (Sun et al., 2022) proposes a style transfer approach that trains a golden subject-specific classifier and transforms other subjects into generators to fit that classifier. The generator is trained by BCE loss and perceptual loss. It is most similar to the proposed method, and the difference between the two methods will be discussed later in the ablation study.
[8] SSSTN (ours) introduces a novel subject-to-subject semantic style transfer network (SSSTN) designed to address the BCI illiteracy problem. Our method incorporates a semantic-aware style transfer loss function, which consists of content, style, and semantic losses to preserve and transfer essential information while improving classification performance. SSSTN consists of three main phases: pretraining, style transfer, prediction and ensemble.

**Table 1:** Performance comparison of proposed and competing methods for subject-dependent scenario on the BCI Competition IV-2a dataset.

**Input-type**	**Method**	**S01**	**S02**	**S03**	**S04**	**S05 (source)**	**S06**	**S07**	**S08**	**S09**	**Mean**
Input-type	EEGNet [1]	78.54	55.14	90.55	55.14	67.71	53.26	84.38	80.69	66.60	70.22
	DeepConvNet [2]	77.40	52.14	85.75	68.39	74.00	59.19	72.96	80.10	80.93	72.32
	DRDA [3]	83.19	55.14	87.43	75.28	62.29	57.15	86.18	83.61	82.00	74.70
	MI [4]	79.51	56.60	89.23	67.36	72.22	60.07	68.06	78.47	79.17	72.30
[-2.5ex]makecell	CWT-CNN [5]	**87.07**	56.17	**92.97**	68.67	39.85	52.00	89.85	72.14	82.56	71.25
	SE-CNN [6]	83.26	53.54	92.57	70.42	68.13	60.90	89.51	83.40	85.63	76.37
	STNN [7]	82.29	47.57	92.57	60.76	66.67	57.99	85.76	77.08	80.90	72.40
	**SSSTN (Ours) [8]**	86.46	**58.33**	92.57	**75.35**	**80.90**	**67.01**	**93.06**	**85.76**	**86.46**	**80.66**

The bold denotes the highest performance in each column.

### 3.2. Experimental settings

Regarding dataset splitting, we used the first of two sessions of the BCI Competition IV-2a dataset as the training set and the second as the test set during the training of SSSTN and all competing methods. For the BCI Competition IV-2b dataset, the first three sessions were used for training, while the remaining two sessions served as test data. During the initial model training, we allocated 10% of the training set for use as a validation set. We then proceeded to tune the hyperparameter configuration based on the model's performance on this validation set. Due to the small size of the dataset, we incorporated the validation set back into the training set for the final model training. This allowed us to leverage the full dataset for training, thereby potentially improving our model's performance.

As previously mentioned, we designated subject 3 as the source subject and assigned the remaining subjects as target subjects for the BCI Competition IV-2a dataset. Similarly, we selected subject 5 as the source subject and allocated the remaining subjects as target subjects for the BCI Competition IV-2b dataset. We evaluated the performance of each model with classification accuracy. All competing methods were trained using the number of epochs and hyperparameters mentioned in each paper. The training process of the proposed SSSTN consists of pretraining and style transfer (since the model is not trained in the prediction and ensemble process). In the pretraining phase, each classifier was trained with a learning rate of 0.0002 for 3,000 epochs. For the style transfer phase, the generator was trained with a learning rate of 0.003 for 600 epochs for the BCI Competition IV-2a dataset. The experimental settings for the 2b dataset were consistent with those of the 2a dataset, with the sole difference being a reduced training duration, set at 200 epochs. Finally, we adopted α as 0.1, β as 1, and γ as 1 for style loss.

### 3.3. Performance evaluations

Table 1 presents the classification accuracy of each method under the subject-dependent scenario in the BCI Competition IV-2a dataset. The proposed SSSTN method demonstrated superior performance with the mean accuracy of 80.66% on the BCI Competition IV-2a dataset, outperforming all other competing methods. The SSSTN also exhibited the highest accuracy on all the individual subjects except subject 3. It is worth noting that since subject 3 was used as the source subject, it did not undergo any additional training beyond the pretraining phase. Among the subjects included in the study, subjects 2 and 6 were considered to be BCI illiterate as they exhibited particularly low classification accuracy in the dataset. Our proposed SSSTN method achieved significant improvements in classification accuracy on both BCI illiterate subjects 2 and 6, compared to other competing methods evaluated on the same dataset. Specifically, the SSSTN method demonstrated notable performance gains over our baseline model, i.e., SE-CNN [6], achieving improvements of 4.79 and 6.11% on subject 2 and subject 6, respectively. Moreover, when compared to the second-best performing method, i.e., MI [4] (signal-based), the SSSTN method continued to demonstrate superiority with improvements of 1.73 and 6.94% on subjects 2 and 6, respectively. Remarkably, the proposed SSSTN method demonstrated substantial improvement in performance for individuals, such as subject 5, who exhibited moderately low classification accuracy in the dataset. To be specific, the SSSTN achieved a significant enhancement of 12.77 and 8.68% in classification accuracy compared to our baseline model and the second-best performing method, respectively.

Among our competing methods, the STNN [7] was considered as the most analogous competitor to our proposed approach due to its comparable implementation of subject-to-subject feature-level style transfer based on source classifiers. The BCE loss used in the STNN can be aligned with our semantic loss in terms of conceptual similarity. However, unlike the perceptual loss function employed in the STNN that calculates the L2 loss between the source feature ($h_{S}^{l}$) and transformed target feature ($h_{S^{\prime }}^{l}$), our style loss function measures the difference between the probability distributions of the semantic styles of $h_{S}^{l}$ and $h_{S^{\prime }}^{l}$ by utilizing KL divergence. Furthermore, the STNN did not have the content loss designed to guarantee the retention of salient information derived from the target. These key differences in loss functions make the STNN a suitable comparison method for our proposed method when exploring the impact of different types of style loss and the absence of content loss. In comparison to the STNN, our SSSTN surpassed its performance across all subjects. Notably, our semantic style loss encourages target and source feature distribution alignment, while STNN's perceptual loss focuses on matching feature values. Experimental results convincingly demonstrated that our proposed style loss contributes to superior classification performance compared to the perceptual loss.

Overall, these findings suggest that our proposed method holds the potential for addressing the BCI illiteracy problem, especially for individuals who were previously difficult to classify MI tasks. In addition to the significant improvements observed on BCI illiterate subjects, our proposed SSSTN method achieved high levels of accuracy on top-performing subjects in the dataset. Particularly, our method exhibited an improvement in the performance of 3.55% on subject 7, when compared to our baseline model. Additionally, our method demonstrated robust performance on subjects with intermediate levels of classification accuracy. In a nutshell, we want to emphasize that our proposed method is effective across a range of BCI proficiency levels, and may have broad applicability for individuals with varying levels of BCI performance.

As illustrated in Table 2, we conducted an additional experiment utilizing the BCI Competition IV-2b dataset. It is essential to note that SE-CNN denotes our baseline network. Following SE-CNN's pretraining, subject 5 achieved the highest performance of 95.83%, serving as the source subject, while subject 2, with a performance of 67.71%, was identified as the BCI illiterate. Without the need for any additional hyperparameter tuning or alterations to network architectures, our SSSTN consistently outperformed the competing methods in terms of mean accuracy in this experiment. Remarkably, SSSTN was the sole method to achieve a classification accuracy exceeding 70% for all subjects, including subject 2, and displayed the highest accuracy for the majority of subjects. The analysis of the BCI Competition IV-2b dataset substantiates that our proposed SSSTN can be effectively applied to a diverse range of datasets, thereby demonstrating the generalizability of our approach.

**Table 2:** Performance comparison of proposed and competing methods for subject-dependent scenario on the BCI Competition IV-2b dataset.

**Input-type**	**Method**	**S01**	**S02**	**S03**	**S04**	**S05 (source)**	**S06**	**S07**	**S08**	**S09**	**Mean**
Input-type	EEGNet [1]	71.10	67.13	74.19	95.02	74.04	71.95	79.89	80.75	80.01	77.19
	DRDA [3]	81.37	62.86	63.63	**95.94**	93.56	**88.19**	85.00	**95.25**	**90.00**	83.98
Input-type	STNN [7]	85.00	**75.00**	68.40	98.90	75.00	82.00	83.20	79.50	79.00	80.70
	SE-CNN [6]	78.12	67.71	83.68	94.44	**95.83**	81.25	90.28	89.93	87.50	85.41
	**SSSTN (Ours) [8]**	**79.58**	70.42	**86.67**	95.42	**95.83**	82.92	**92.08**	90.00	**90.00**	**86.99**

The bold denotes the highest performance in each column.

### 3.4. Ablation study

We performed an ablation study on the BCI Competition IV-2a to validate the effectiveness of our proposed method and its loss functions. Table 3 presents the variants of our SSSTN method utilized in this ablation study. Specifically, SSSTN-A represents the model that excludes the content loss, SSSTN-B is the model that omits the style loss, and SSSTN-C indicates the model without the semantic loss. Finally, SSSTN refers to the entire network we proposed. The remaining SSSTN and its variants all achieved higher mean accuracy than SE-CNN. These results suggest that our proposed SSSTN method with different types of losses has a positive impact on the classification performance of the model. In terms of mean accuracy, the SSSTN-A exhibited the most inferior performance, succeeded by SSSTN-B, subsequently, SSSTN-C. From these observations, we conclude that content loss, followed by style loss and semantic loss, contributes to improved classification performance. It is worth noting that our SSSTN method outperformed all other methods, displaying the highest performance improvement across nearly all subjects. Specifically, for subjects 2, 5, and 6, classified as BCI illiterate due to low classification accuracy, the SSSTN-B model exhibited the most significant performance improvement, surpassing all other models except for the complete SSSTN method. This outcome suggests that the content loss is one of the most critical factors in addressing the BCI illiteracy issue because it helps to retain the class-relevant feature representation of *x*_*T*_. As demonstrated in Section 3.3.1, our proposed style loss exhibited superior performance, which becomes evident when comparing the results of STNN and our SSSTN. Therefore, these findings demonstrate the effectiveness of our proposed SSSTN method in addressing the BCI illiteracy problem, which is achieved through leveraging classifier-based feature-level semantic style transfer with appropriate loss functions.

**Table 3:** Ablation study results demonstrating the impact of removing specific components from the proposed SSSTN method on the BCI Competition IV-2a dataset.

**Method**	**S01**	**S02**	**S03**	**S04**	**S05**	**S06**	**S07**	**S08**	**S09**	**Mean**
SE-CNN [6]	83.26	53.54	92.57	70.42	68.13	60.90	89.51	83.4	85.63	76.37
SSSTN-A (w/o $L_{\text{cont}}$)	81.60	53.47	92.57	68.06	74.65	65.62	90.28	85.42	83.68	77.26
SSSTN-B (w/o $L_{\text{style}}$)	84.36	53.82	92.57	72.57	75.00	**68.75**	92.01	84.03	86.11	78.80
SSSTN-C (w/o $L_{\text{sem}}$)	84.03	52.78	92.57	74.31	75.00	67.01	**93.06**	**85.76**	**86.81**	79.04
**SSSTN (Ours) [8]**	**86.46**	**58.33**	92.57	**75.35**	**80.90**	67.01	**93.06**	**85.76**	86.46	**80.66**

The bold denotes the highest performance in each column.

In order to ascertain that our SSSTN can be adaptable to different source subjects, we conducted additional experiments on the BCI Competition IV-2a to validate this claim by adopting subject 7, which had the second-highest accuracy of 89.51% among the subjects in our baseline network (SE-CNN), as the source subject. As shown in Table 4, the experimental results demonstrated that our proposed method is not confined to a specific source subject, i.e., subject 3, but also exhibits robust performance when applied to subject 7, thereby confirming its adaptability and applicability to various source subjects. Employing subject 7 as the source subject, the SSSTN achieved a mean accuracy of 78.74%, surpassing the performance of the baseline network. However, it is worth mentioning that selecting subject 7 as the source subject led to a minor improvement in performance from the baseline compared to when subject 3 was chosen, which can be attributed to the relatively lower classification accuracy of subject 7 in relation to subject 3.

**Table 4:** Additional results demonstrating the applicability of our SSSTN model to other source subjects beyond the single source subject on the BCI Competition IV-2a dataset.

**Method**	**S01**	**S02**	**S03**	**S04**	**S05**	**S06**	**S07**	**S08**	**S09**	**Mean**
SE-CNN [6]	83.26	53.54	92.57	70.42	68.13	60.90	89.51	83.4	85.63	76.37
SSSTN (Source:3)	**86.46**	**58.33**	92.57	**75.35**	**80.90**	67.01	**93.06**	**85.76**	**86.46**	**80.66**
SSSTN (Source:7)	81.60	54.86	**95.49**	73.61	76.39	**70.83**	89.51	85.07	85.07	78.74

The bold denotes the highest performance in each column.

## 4. Discussion

### 4.1. t-SNE visualization

**Figure 4:**
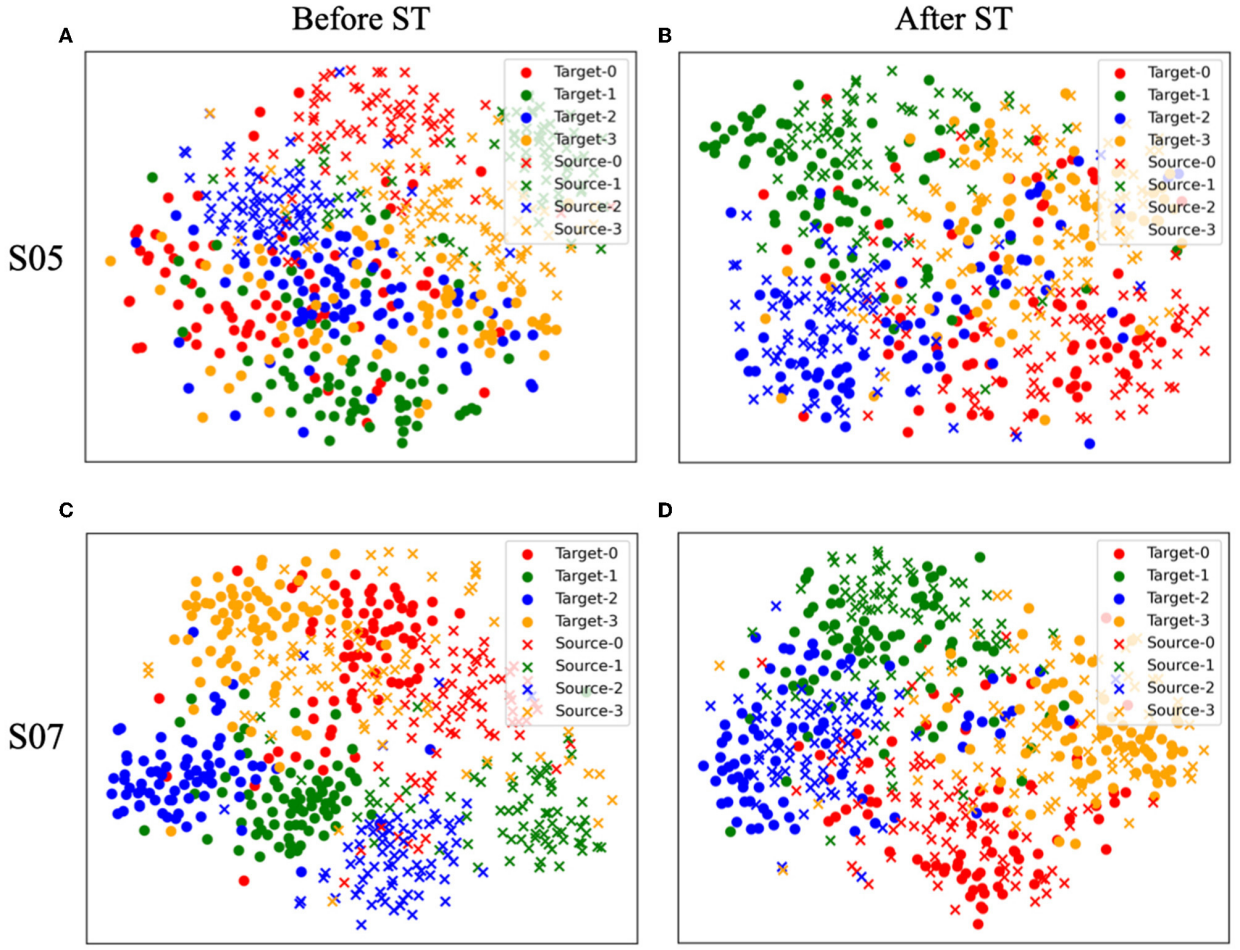
t-SNE (Van der Maaten and Hinton, 2008) visualization of the change in the h^2^ feature distribution of the target (S05, S07) and source (S03) before and after the style transfer. **(A, B)** Before/after style transfer from S05 to source. **(C, D)** Before/after style transfer from S07 to source.

To examine the impact of style transfer, we employed the t-SNE algorithm (Van der Maaten and Hinton, 2008) to visualize the feature *h*^2^ obtained from the second convolutional layer of *C* before and after the transformation. This experiment was carried out on a test set of Subjects 5 and 7 that were randomly selected. Figures 4A, C depict $h_{T}^{2}$ and $h_{S}^{2}$ before style transfer (ST), while Figures 4B, D show $h_{S^{\prime }}^{2}$ and $h_{S}^{2}$ after ST, both in two-dimensional embedding space. Each marker represents the subject (source or target) of the samples, and each color corresponds to the label of the samples. Before the transformation, distinct distributions were observed between the target and source subjects, evident for both Subjects 5 and 7 as illustrated in Figures 4A, C. However, after transformation, the target and source distributions merged into a single distribution, as depicted in Figures 4B, D. Note that the same labels are consistently clustered together, regardless of whether they belong to the source or target data. This result supports the idea that our proposed style transfer process effectively preserved the underlying class-relevant feature representation. In particular, before the transformation of Subject 5, the target data was not distinctly separated by the label as shown in Figure 4A. However, as shown in Figure 4B, after the transformation, the target data exhibited a significantly better separation by the label and were closely clustered with the source data samples that shared the same label. The obtained results suggest that the proposed SSSTN method effectively improves the discriminability of the transformed target features, leading to better classification by *C*_*S*_. Therefore, we verified that the proposed SSSTN method transfers the target data to a well-classified feature space of the source data, while preserving the feature class-relevant representation of the target data.

### 4.2. Training loss analysis

**Figure 5:**
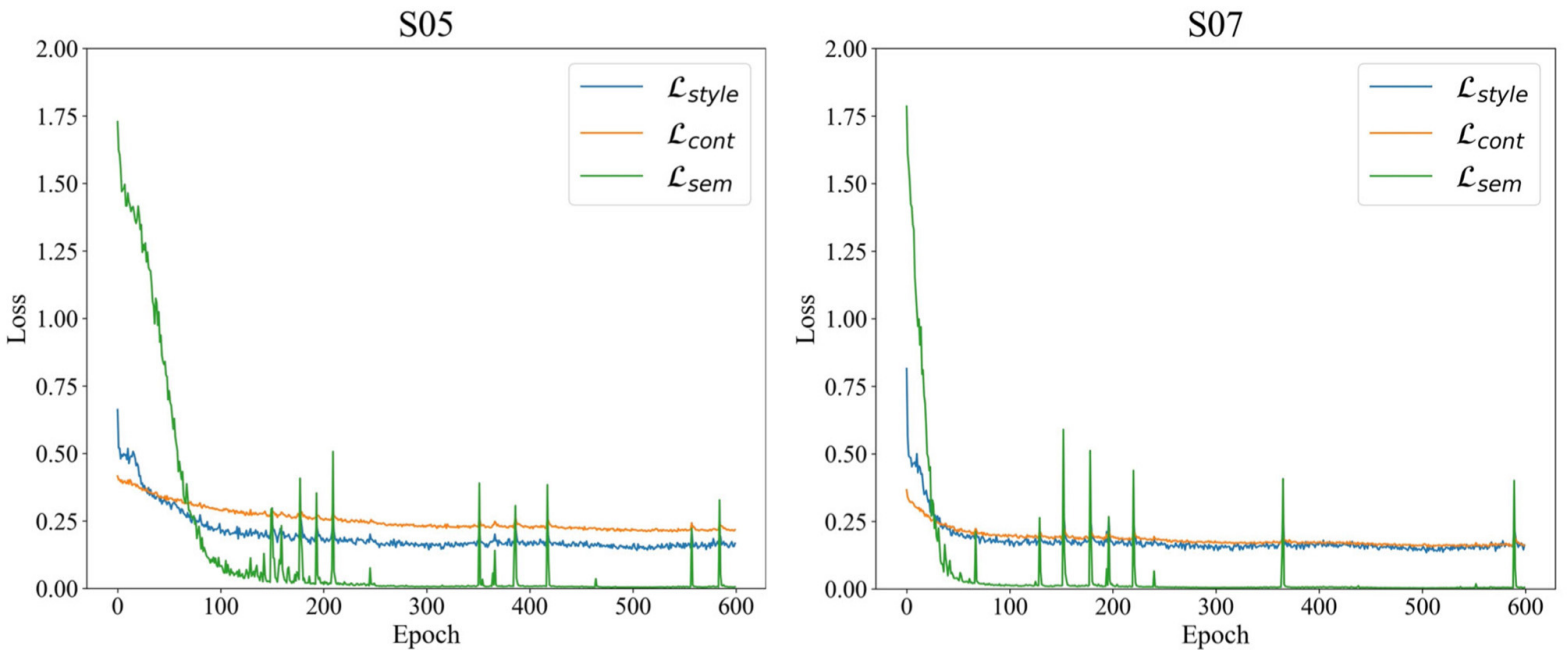
Training loss plot of the SSSTN for subject 5 and 7. L_*style*_, L_*cont*_, and L_*sem*_ denote style, content, and semantic loss, respectively.

As part of our analysis of the SSSTN network, we plotted the training loss graphs for subjects 5 and 7, which show the changes in style, content, and semantic loss over time. It is noteworthy that our selection of subjects 5 and 7 is consistent with our t-SNE visualization analysis. As depicted in Figure 5, the loss converges to a stable value after approximately 600 epochs of training. This convergence demonstrates the stability of the SSSTN during the training process and provides a profound understanding of the network's overall performance.

### 4.3. Practical potential of our proposed method

In this section, we investigate the real-world application and the practical potential of our proposed method. Our proposed method is based on the assumption that we can identify a BCI expert (source subject) and BCI illiterates (target subjects) through a pretrained classification network. As mentioned in the introduction and supported by prior studies (Zhang et al., 2020; Wang et al., 2021), subjects with performance below 70% for a predefined period are classified as BCI illiterates. Our study seeks to enhance the performance of these illiterate subjects using a pretrained network. In this context, it could be feasible to distinguish between a BCI expert, who exhibits the highest classification performance, and BCI illiterates who score less than 70% based on the pretrained network. We then use a semantic style transfer process to transform the BCI illiterates' data into the expert's style, thereby improving their performance further. We believe that this approach offers potential for real-world applications in situations where an existing BCI system, specifically a pretrained classifier, is available to effectively distinguish between BCI expert and BCI illiterate subjects. Furthermore, our proposed method relies on prior knowledge, such as a pretrained network, but is not restricted to a specific network. This flexibility allows for its application to networks with varying architectures. We will continue to investigate strategies to enhance the generalizability and practicality of our method in future studies.

## 5. Conclusion

In this study, we proposed a subject-to-subject semantic style transfer network (SSSTN) to address the problem of BCI illiteracy in EEG-based motor imagery classification tasks. The proposed SSSTN leverages subject-specific classifier-based modified style loss and content loss to effectively transfer invisible feature-level semantic styles from source subject (BCI expert) to target subjects (specifically, BCI illiterates) while preserving their class-relevant semantic information of target subjects. Therefore, the transformed data from the target to the source retains the distribution of class-discriminative features from the source, leading to better classification performance by the source classifier. Experimental results on the BCI Competition IV-2a dataset show that our proposed method outperforms other competing methods, especially for the BCI illiterate. The ablation study and t-SNE visualization demonstrate the ability to achieve meaningful feature-level semantic style transitions by confirming the effectiveness of each component within the SSSTN. Furthermore, the ensemble approach used in this method contributes to improving classification performance by fusing different feature representations. This study paves the way for further research on subject-to-subject style transfer and BCI illiteracy mitigation.

## Data availability statement

The original contributions presented in the study are included in the article/supplementary material, further inquiries can be directed to the corresponding author.

## Author contributions

D-HK and D-HS designed the research and wrote the initial manuscript. D-HK designed and conducted the experiments and analyzed the results. D-HS preprocessed the data. T-EK supervised the research and revised the manuscript. All authors contributed to the article and approved the submitted version.
